# Manual muscle testing and hand-held dynamometry in people with inflammatory myopathy: An intra- and interrater reliability and validity study

**DOI:** 10.1371/journal.pone.0194531

**Published:** 2018-03-29

**Authors:** Pierrette Baschung Pfister, Eling D. de Bruin, Iris Sterkele, Britta Maurer, Rob A. de Bie, Ruud H. Knols

**Affiliations:** 1 Directorate of Research and Education, Physiotherapy Occupational Therapy Research Center, University Hospital Zurich, Zurich, Switzerland; 2 Department of Health, Institute of Physiotherapy, Zurich University of Applied Sciences, Winterthur, Switzerland; 3 Functioning and Rehabilitation, CAPHRI Care and Public Health Research Institute, Maastricht University, 6200 MD Maastricht, The Netherlands; 4 Department of Health Sciences and Technology, Institute of Human Movement Sciences and Sport, ETH Zurich, Zurich, Switzerland; 5 Division of Physiotherapy, Department of Neurobiology, Care Sciences and Society, Karolinska Institutet, SE141 83 Huddinge, Sweden; 6 Nursing and Allied Health Professions Office, Physiotherapy Occupational Therapy, University Hospital Zurich, Zurich, Switzerland; 7 Department of Rheumatology, University Hospital Zurich, Zurich, Switzerland; 8 Department of Epidemiology, CAPHRI Care and Public Health Research Institute, Maastricht University, 6200 MD Maastricht, The Netherlands; University of Illinois at Urbana-Champaign, UNITED STATES

## Abstract

Manual muscle testing (MMT) and hand-held dynamometry (HHD) are commonly used in people with inflammatory myopathy (IM), but their clinimetric properties have not yet been sufficiently studied. To evaluate the reliability and validity of MMT and HHD, maximum isometric strength was measured in eight muscle groups across three measurement events. To evaluate reliability of HHD, intra-class correlation coefficients (ICC), the standard error of measurements (SEM) and smallest detectable changes (SDC) were calculated. To measure reliability of MMT linear Cohen`s Kappa was computed for single muscle groups and ICC for total score. Additionally, correlations between MMT8 and HHD were evaluated with Spearman Correlation Coefficients. Fifty people with myositis (56±14 years, 76% female) were included in the study. Intra-and interrater reliability of HHD yielded excellent ICCs (0.75–0.97) for all muscle groups, except for interrater reliability of ankle extension (0.61). The corresponding SEMs% ranged from 8 to 28% and the SDCs% from 23 to 65%. MMT8 total score revealed excellent intra-and interrater reliability (ICC>0.9). Intrarater reliability of single muscle groups was substantial for shoulder and hip abduction, elbow and neck flexion, and hip extension (0.64–0.69); moderate for wrist (0.53) and knee extension (0.49) and fair for ankle extension (0.35). Interrater reliability was moderate for neck flexion (0.54) and hip abduction (0.44); fair for shoulder abduction, elbow flexion, wrist and ankle extension (0.20–0.33); and slight for knee extension (0.08). Correlations between the two tests were low for wrist, knee, ankle, and hip extension; moderate for elbow flexion, neck flexion and hip abduction; and good for shoulder abduction. In conclusion, the MMT8 total score is a reliable assessment to consider general muscle weakness in people with myositis but not for single muscle groups. In contrast, our results confirm that HHD can be recommended to evaluate strength of single muscle groups.

## Introduction

Inflammatory myopathies (IMs), including dermatomyositis (DM), polymyositis (PM) and associated myopathies, are systemic rheumatic muscle diseases characterized by chronic muscle inflammation [1]. With a worldwide average annual incidence rate ranging from 0.2 to 8 in 100`0000, IMs are relatively rare. However, the burden of the disease for affected patients is considerable. The most prominent clinical features in IM are muscle weakness and low muscle endurance, which progresses over a period of weeks or months [2, 3]. Most commonly, the weakness is symmetrical, proximal extremity muscles appear to be more affected, and neck flexors are weaker than extensors [4]. As a consequence of muscle weakness, people with myositis often report difficulties with activities of daily living, e.g., getting up from a chair, going up- or down the stairs, getting into a car, stepping onto a curb, lifting objects, washing hair, brushing teeth, and gripping objects [1, 2].

The International Myositis Assessment and Clinical Study Group (IMACS) defines muscle strength as one of the core outcomes to be measured for assessing myositis disease activity and damage [5]. As the limb-girdle muscles and anterior neck flexors are among the leading indicators of myositis [6], the measurement of muscle strength in these muscle groups is a relevant parameter when diagnosing IM. Furthermore, muscle strength should also be used as an intervention outcome to evaluate the effects of progressive resistance training in rehabilitation programs. The foregoing emphasizes the necessity for a widely-accepted assessment to measure muscle strength in people with myositis. To date, however, there is no consensus about the most accurate way to assess muscle strength in this patient group.

Manual muscle testing (MMT), which is scored using a 0–5 point Medical Research Council muscle strength scale or a 0–10 point Kendall grading scale and hand-held dynamometry (HHD) which measures the peak isometric force generated from a muscle group, are two common methods to assess muscle strength in therapeutic IM trials [7]. MMT is less time consuming and, therefore, less stressful for people with myositis but has decreased sensitivity and specificity in detecting mild weakness and exhibits ceiling effects. Furthermore, the grading system of MMT is subjective and varies with the strength of the examiner [5, 8]. Despite this deficiency, the MMT8, a myositis specific subset of MMT, is the most frequently used assessment in myositis trials [5, 7]. HHD is used less commonly than MMT, but being an objective measure of muscle strength, it has the potential to overcome some of the limitations of the latter. HHD may detect mild deteriorations or improvements during the course of IM or after a resistive strength training program [5]. These low-cost and portable devices assess muscle strength reliably in clinical settings when using specific procedures [9]. Furthermore, HHD has demonstrated to have good concurrent validity when compared with laboratory-based isokinetic dynamometry testing [10–12]. Although MMT and HHD are both supposed to measure muscle weakness, reports about the relationship between the two methods show conflicting results. Whilst some authors concluded that these methods measure the same construct [13, 14], other authors indicated no clear relationships between MMT and HHD [15, 16].

Clinimetric properties, in particular reliability and concordance between the MMT8 and the HHD, have never been conclusively determined in people with myositis. Two studies, each with seven patients, investigated the relative reliability of the MMT8 and the HHD. ICCs between 0.28 and 0.85 were reported for the MMT8 whilst reliability values of the HHD ranged from 0.88 to 0.98 [4, 17]. Absolute agreement parameters have, to the best of our knowledge, not been reported. Although both measures (MMT8 and HHD) are used to assess maximal voluntary isometric muscle contraction it is not investigated whether the results of MMT8 and HHD in people with myositis are comparable.

The first aim of the present study was, therefore, to evaluate intra- and interrater reliability of the MMT8 and HHD in adults with myositis. Secondly, this study aimed to determine concordance between MMT8 and HHD. It was hypothesised that HHD would demonstrate excellent reliability (ICC>0.75), that MMT8 would demonstrate substantial reliability (Kappa values between 0.61 and 0.8) and that the concordance between HHD and MMT8 would be good (Spearman correlation between 0.7 and 0.9) for all tested muscle groups.

## Material and methods

### Participants

A convenience sample of 50 people with myositis was recruited from the Department of Rheumatology of the University Hospital Zurich, Switzerland between August 2014 and May 2016. All patients presenting for evaluation of myositis were asked by their physician if they would be interested to participate in this study. Interested patients were then contacted by one of the researchers and checked for inclusion and exclusion criteria. Inclusion criteria were diagnosis of polymyositis, dermatomyositis or a myositis associated disorder (scleroderma, systematic lupus erythematosus, Sjögren`s syndrome), age over 18, and ability to read and understand German. Exclusion criteria were diagnoses of inclusion body myositis, pulmonary hypertension, osteoporosis, severe cardiovascular and/or pulmonary disease, pain syndrome, and paresis. The participants gave their signed informed consent to participate, and the study was approved by the local ethics committee (registration no. 2014–0022 of the Cantonal Ethics Committee Zurich, Switzerland). The individual in this manuscript demonstrating a measurement set-up has given written informed consent (as outlined in PLOS consent form) to publish these case details. This study is registered at the ClinicalTrials.gov (registration number: NCT03059394).

**Fig 1:**
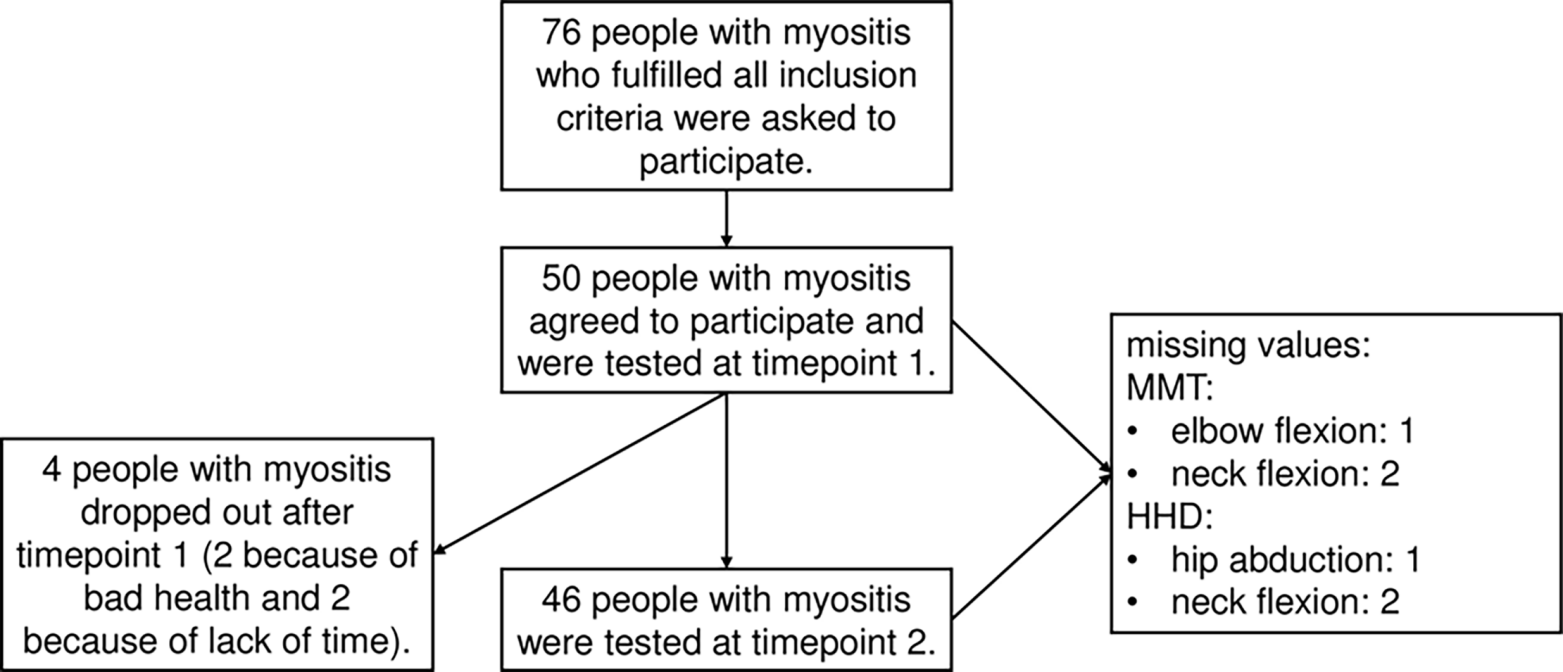
Sample selection. From 76 people with myositis fulfilling all inclusion criteria 50 agreed to participate. After the first measurement four participants dropped out.

Out of 76 people with myositis who met all inclusion criteria, 50 agreed to participate. Four dropped out after the first measurements. Therefore, reliability was analyzed with data from 46 participants. Due to pain or incapacity to perform certain test positions, some muscle groups could not be tested in all participants. The detailed sample selection process is shown in Fig 1.

### Testers

The measurements were performed by two senior physiotherapists, experienced with treatment and measurement of people with rheumatologic diseases. The female physiotherapists were 35 and 47 years old, had a body height of 162 cm and 175 cm and weighted 49 kg and 60 kg, respectively. The two testers were instructed and trained in the use of the MMT8 and the HHD before study start.

### Procedures

**Fig 2:**
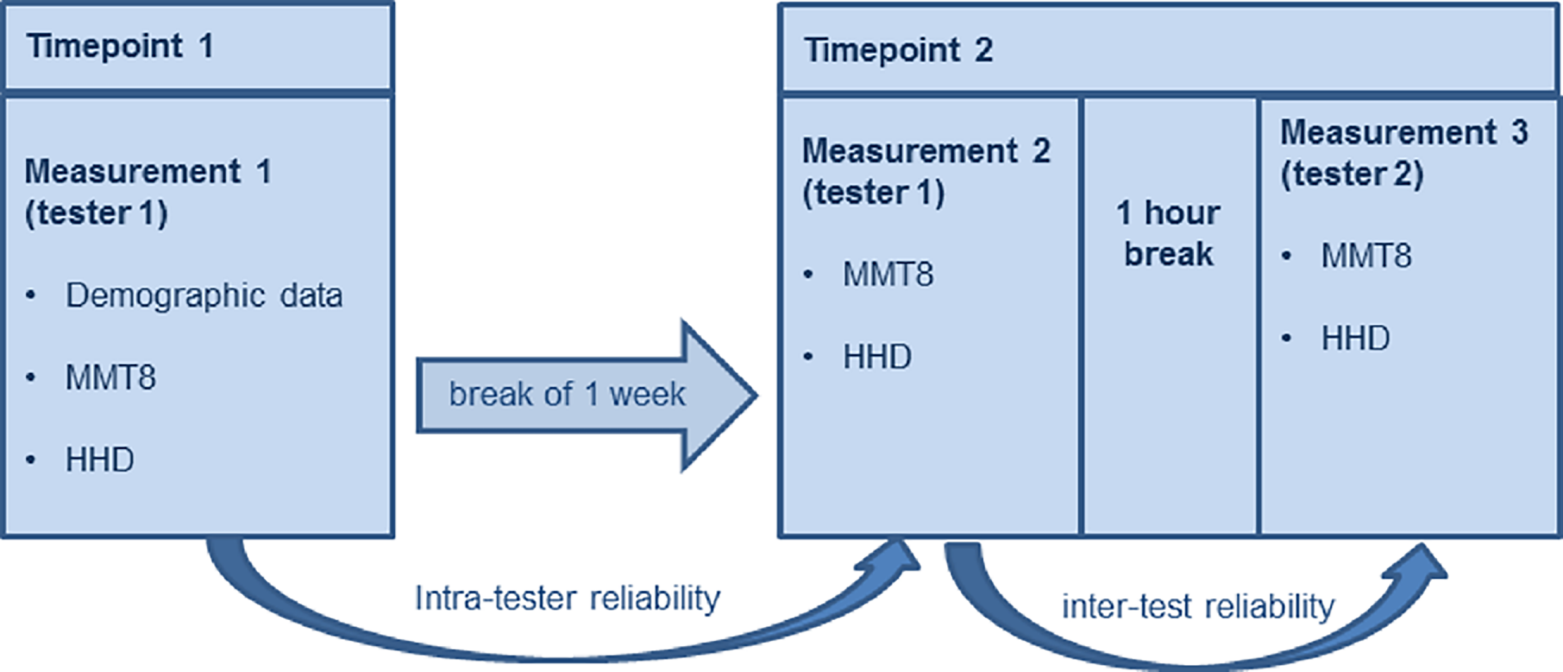
Procedure of the intra- and interrater reliability. Each patient was measured three times. For intrarater reliability MMT8 and HHD were performed by the same tester (Measurement 1 and 2) and for interrater reliability MMT8 and HHD were performed by two different testers (Measurement 2 and 3). Abbreviations: MMT8: Manual muscle test 8, HHD: Hand-held dynamometry.

Each participant was measured three times. At time point 1 demographic data (gender, age, BMI, diagnosis, disease stage, time since diagnosis) were collected and tester 1 conducted the MMT8 and the HHD (Measurement 1). For intrarater reliability, at time point 2 (one week later), MMT8 and HHD were performed by the same tester (Measurement 2). After a one-hour break, the MMT8 and HHD were conducted by tester 2 for interrater reliability (Measurement 3, Fig 2). Measures were performed in the same order, in the same test room and if possible at the same time of the day, to optimize the standardisation of the test procedure.

#### Manual muscle testing (MMT8)

The dominant side of the following eight muscle groups was tested in a standardised order: shoulder abduction, elbow flexion, ankle extension, hip abduction, hip extension, knee extension, wrist extension and neck flexion. The dominant side was based on the self-declared hand preference. Detailed description of the participants’ and therapist`s position and the precise test instructions for each muscle group is described in the “manual muscle testing procedure for MMT8 Testing”. Each muscle group was scored according the Kendall 10-point Scale (Table 1) [18]. Scores between 0–3, 4–6, and 7–9 indicate severe, moderate and mild weakness, respectively and a score of 10 means that there is no detectable weakness [19]. The single scores were added to receive a total score varying from 0 to 80 (0 = no muscle contraction, 80 = normal strength).

**Table 1:** Kendall 10-point scale.

test procedure	function of the muscle	raw score	graded scored
**no movement**	No visible movement of the part	0	severe weakness
**test movement**	**Movement in horizontal plane**		
	Moves through partial range of motion	1	
	Moves through complete range of motion	2	
	**Movement against gravity**		
	Moves through partial range of motion	3	
**test position**	gradual release from test position	4	moderate weakness
	Holds test position (no added pressure)	5	
	Holds test position against slight pressure	6	
	Holds test position against slight to moderate pressure	7	mild weakness
	Holds test position against moderate pressure	8	
	Holds test position against moderate to strong pressure	9	
	Holds test position against strong pressure	10	no weakness

#### Hand-held dynamometry

Muscle strength of the same muscle groups that were included in the MMT8 was assessed using the MicroFET2 hand-held dynamometer. The MicroFET2 is a battery operated hand-held device which measures peak force in Newtons (N), up to a value of 890N (Force Evaluating and Testing, Hoggan Health Industries Inc. West Draper, UT, USA). Each muscle action was measured in a gravity-neutralized position. Testing procedure and test position were performed according to standardised protocols [20–22]. After at least one familiarization trial, each muscle group was assessed twice. Isometric “make” tests were used [20]. Peak force values were recorded for each trial. Participant position, placement of the dynamometer, verbal instruction and location of stabilisation provided for each tested muscle group are described in the “Manual Quantitative Muscle Testing”. The individual in this manuscript demonstrating a measurement set-up has given written informed consent (as outlined in PLOS consent form) to publish these case details.

### Data analysis

Demographic data (gender, age, BMI, diagnosis, disease stage, time since diagnosis) were defined using descriptive statistics. Normality of the data was evaluated using Shapiro Wilk test. The level of significance was set to α≤0.05 (with Bonferroni correction for multiple comparisons). No imputation was performed. A case was deleted when a variable was missing for a particular analysis, however, this case was included in analyses for which all required variables were present. Due to this pairwise deletion, the total N was not consistent across all analyses. SPSS version 22.0 (SPSS Inc, Chicago, Illinois) was used for data analysis.

#### Hand-held dynamometry

Data of each muscle group and total score were summarized by mean and standard deviation. To compute total scores, the values of each muscle group were added and this sum was divided by eight. The peak force of the best trial (peak force) and the averaged force of the two performed trials (averaged peak force) were used for data analysis.

Relative reliability, which expresses how well participants can be distinguished from each other despite the presence of measurement error, was determined by calculating intraclass correlation coefficient (ICC) [23]. The ICC_2_ (A,1) formula for reliability of the highest score and the ICC_2_ (A,k) formula for reliability of the average score were used [23, 24]. For interpretation of ICC values, the following classification was considered: >0.75 excellent reliability, 0.40–0.75: fair to good reliability, and <0.40 poor reliability [25].

To evaluate changes over time, variability between participants and, therefore, relative reliability is not particularly informative. In this case absolute measurement error, also called agreement parameters, are indicated [26]. Therefore, the standard error of measurement (SEM) and the smallest detectable change (SDC) were calculated. The SEM represents the standard deviation of repeated measures of one individual and is calculated with the formula SEM_agreement_ = √(σ_pt_^2 +^ σ_resiudual_^2^) [26]. The SDC represents the minimal change that must be overcome to ensure real change and is calculated with the formula SDC = 1.96 x √2 x SEM [26]. To evaluate a systematic failure between strength measures, Bland an Altman plots were drawn with the free Medcalc statistic software (MedCalc Software, Ostend, Belgium) [27].

#### Manual muscle testing (MMT8)

Raw MMT scores (0–10) as well as graded MMT scores (0–3: severe weakness, 4–6: moderate weakness, 7–9: mild weakness, 10: no weakness) [19] are ordinal scales and, therefore, summarized by medians and interquartile ranges for single muscle groups and for the graded total score. Floor and ceiling effects were determined by calculating the number of individuals obtaining, respectively, the highest or lowest scores, where a limit of 15% should not be exceeded [28].

To measure reliability of single muscle groups and for the graded total score weighted Cohen`s Kappa was computed using the GraphPad software (http://faculty.vassar.edu/lowry/kappa.html). Because misclassifications between adjacent categories are less serious than those between more distant categories we used a linear Kappa [23]. To interpret kappa values we applied Landis and Koch benchmarks (>0.8: almost perfect, 0.61–0.8: substantial, 0.41–0.6: moderate, 0.21–0.4: fair, <0.2 slight) [29]. For ordinal data there are no parameters of measurement error that quantify the measurement error in units of measurement [23].

Raw MMT total scores are summarized as means and standard deviations and parametric statistics was used because they approximated interval data. Reliability of the raw total score was determined by calculating intraclass correlation coefficient (ICC_2_ (A,1), SEM and SDC.

#### Concordance between HHD and MMT8

Correlations between HHD and MMT8 were calculated with Spearman`s rho. When scoring the MMT, raters might consider participant`s body weight. Therefore, absolute force as well as normalized force (absolute force divided by body weight) of HHD were correlated with the MMT. A Spearman correlation coefficient greater than 0.9 was considered ‘excellent’, a coefficient between 0.7 and 0.9 ‘good‘ and one between 0.5 and 0.7 ‘moderate‘ [30]. Additionally, the associations between the two muscle strength assessments are depicted with boxplots with strength values of each muscle group displayed for the MMT grades.

## Results

The demographic and health related data of the 50 participants are summarized in Table 2.

**Table 2:** Demographic and health related data of the participants (n = 50).

Characteristics		Values
Sex, n (%)	female	38 (76)
	male	12 (24)
Age, in years	mean ±SD	56 ± 14
	range	21–82
BMI, in kg/cm^2^	mean ±SD	26 ± 5
Diagnosis, n (%)	DM	22 (44)
	PM	17 (34)
	Associated	11 (22)
Disease stage, n (%)	Acute (0–3 weeks after an active disease stage)	9 (18)
	Subacute (between 3 and 12 weeks after an active disease stage)	9 (18)
	Chronic (more 12 weeks after an active disease stage)	32 (64)
Time since diagnosis (months)	mean ±SD	36 ± 18
	range	0.25–240

n: number, SD: standard deviation, DM: Dermatomyositits, PM: Polymyositis

### Hand-held dynamometry

The muscle strength values (M1, M2, and M3) and the reliability parameters (ICC, SEM and SDC) for peak force are presented in Table 3. The mean peak forces ranged from 55 N (wrist extension) to 219 N (knee extension) and the standard deviations ranged from 25 (neck flexion) to 92 N (knee extension). All strength measurement data were normally distributed and there was no significant difference between measurement 1 and 2 or between 2 and 3 (t-test, p≥0.003; corrected for 16 comparisons). For all muscle groups, except for elbow flexion and for knee extension, intrarater reliability of peak force (ICCs between 0.71 and 0.86) was higher than interrater reliability (ICCs between 0.45 and 0.9). For elbow flexion and knee extension, the ICCs for intrarater reliability were lower than those for interrater reliability (0.83 versus 0.9, and 0.82 versus 0.87, respectively). Six out of eight measured muscle groups showed excellent intrarater reliability. Hip abduction and neck flexion had fair to good intrarater reliability. Interrater reliability was excellent for three muscle groups (shoulder abduction, elbow flexion, and knee extension) and fair to good for the other five muscle groups (ankle extension, hip abduction, hip extension, wrist extension and neck flexion). Intra- and interrater reliability was excellent for total score (0.92 and 0.94). The corresponding SEMs for single scores varied between 12 and 37 Newton and the SDCs% ranged from 40 to 70% for intra- and from 33 to 78% for interrater reliability. The SEM for the total score was 12 N and the SDC 27% for intrarater reliability and 10 N and 23% for interrater reliability.

**Table 3:** Peak force at M1, M2 and M3 plus intra- and interrater reliability measured with hand-held dynamometry (N = 46).

	M 1	M 2	M 3	intrarater reliability			interrater reliability		
muscle groups	Newton mean ± SD	Newton mean ± SD	Newton mean ± SD	ICC (95%CI)	SEM (SEM%)	SDC (SDC%)	ICC (95%CI)	SEM (SEM%)	SDC (SDC%)
S_ABD	97 ± 43	92 ± 36	95 ± 43	0.86^++^ (0.75–0.92)	15 (16)	41 (43)	0.78^++^ (0.64–0.87)	19 (20)	52 (55)
E_FLEX	131 ± 57	133 ±49	128 ± 50	0.83^++^ (0.91–0.83)	22 (17)	61 (46)	0.90^++^ (0.83–0.95)	15 (12)	43 (33)
A_EXT	144 ± 47	142 ± 51	128 ± 47	0.82^++^ (0.70–0.90)	21 (14)	57 (40)	0.45^+^ (0.19–0.65)	36 (27)	101 (75)
H_ABD	123±46	122 ± 42*	133 ± 53	0.71^+^ (0.53–0.83)	23 (19)	65 (53)	0.61^+^ (0.40–0.77)	29 (23)	81 (64)
H_EXT	143 ± 59	143 ± 65*	151 ± 60*	0.83^++^ (0.72–0.91)	25 (18)	70 (49)	0.65^+^ (0.44–0.79)	37 (25)	104 (71)
K_EXT	216 ± 86	210 ± 89	219 ± 92	0.82^++^ (0.69–0.90)	37 (17)	103 (48)	0.87^++^ (0.78–0.93)	32 (15)	90 (42)
W_EXT	55 ± 28	60 ± 30	60 ± 31	0.83^++^ (0.72–0.91)	12 (21)	33 (57)	0.69^+^ (0.51–0.82)	17 (28)	47 (78)
N_FLEX	58 ± 28	68 ±33*	62 ± 25**	0.72^+^ (0.51–0.85)	16 (25)	44 (70)	0.65^+^ (0.44–0.79)	17 (26)	47 (73)
total score	121 ± 40	122 ± 41	124 ± 43***	0.92^++^ (0.85–0.96)	12 (10)	32 (27)	0.94^++^ (0.89–0.97)	10 (8)	29 (23)

M: measurement, SD: standard deviation, 95%CI: 95% confidence interval, ICC: Intraclass correlation coefficient, SEM: standard error of measurement, SDC; smallest detectable change, S_ABD: shoulder abduction, E_FLEX: elbow flexion, A_EXT: ankle extension, H_ABD: hip abduction, H_EXT: hip extension, K_EXT: knee extension, W_EXT: wrist extension, N_FLEX: neck flexion

*1 missing values

** 2 missing values

***3 missing values

^+^fair to good reliability

^++^ excellent reliability

The results and reliability parameters of averaged peak force are shown in Table 4. Intrarater and interrater ICCs for single muscle groups and for the total score were excellent (0.75–0.97), except for interrater reliability of ankle extension (0.61) which was fair to good. All SEMs (8-30N) and SDCs% (23–65%) for single muscle groups and for the total score (SEM: 7–8, SDC%: 16–19) were smaller for averaged peak force than for peak force values.

**Table 4:** Averaged peak force at M1, M2 and M3 plus intra- and interrater reliability measured with hand-held dynamometry (N = 46).

	M 1	M 2	M 3	intrarater reliability			interrater reliability		
muscle groups	Newton mean ± SD	Newton mean ± SD	Newton mean ± SD	ICC (95%CI)	SEM (SEM%)	SDC (SDC%)	ICC (95%CI)	SEM (SEM%)	SDC (SDC%)
S_ABD	92 ± 39	88 ± 35	89 ± 42	0.94^++^ (0.87–0.97)	9 (11)	26 (29)	0.88^++^ (0.78–0.93)	13 (15)	37 (42)
E_FLEX	125 ± 53	128 ± 47	123 ± 47	0.90^++^ (0.82–0.95)	16 (12)	43 (34)	0.95^++^ (0.91–0.97)	10 (8)	29 (23)
A_EXT	135 ± 45	136 ± 51	122 ± 46	0.91^++^ (0.84–0.95)	14 (11)	40 (29)	0.61^+^ (0.31–0.78)	30 (23)	84 (65)
H_ABD	114 ± 40	114 ± 37*	125 ± 51	0.84^++^ (0.70–0.91)	16 (14)	43 (38)	0.75^++^ (0.55–0.86)	22 (18)	61 (51)
H_EXT	134.3 ± 58	134 ± 61*	141 ± 57*	0.91^++^ (0.83–0.95)	18 (13)	50 (37)	0.77^++^ (0.58–0.87)	28 (20)	78 (57)
K_EXT	205 ± 83	199 ± 85	207 ± 88	0.92^++^ (0.85–0.95)	24 (12)	68 (33)	0.94^++^ (0.89–0.97)	21 (10)	58 (29)
W_EXT	52 ± 27	55 ± 27	56 ± 29	0.92^++^ (0.86–0.96)	8 (14)	21 (39)	0.84^++^ (0.72–0.91)	11 (20)	31 (55)
N_FLEX	54 ± 25	63 ± 29*	59 ± 24**	0.87^++^ (0.72–0.93)	10 (17)	27 (46)	0.82^++^ (0.66–0.90)	11 (19)	31 (52)
total score	114 ± 38	115 ± 39	117 ± 41***	0.96^++^ (0.92–0.98)	8 (7)	21 (19)	0.97^++^ (0.94–0.98)	7 (6)	19 (16)

M: measurement, SD: standard deviation, 95%CI: 95% confidence interval, ICC: Intraclass correlation coefficient, SEM: standard error of measurement, SDC; smallest detectable change, S_ABD: shoulder abduction, E_FLEX: elbow flexion, A_EXT: ankle extension, H_ABD: hip abduction, H_EXT: hip extension, K_EXT: knee extension, W_EXT: wrist extension, N_FLEX: neck flexion

*1 missing values

** 2 missing values

***3 missing values

^+^fair to good reliability

^++^ excellent reliability

**Fig 3:**
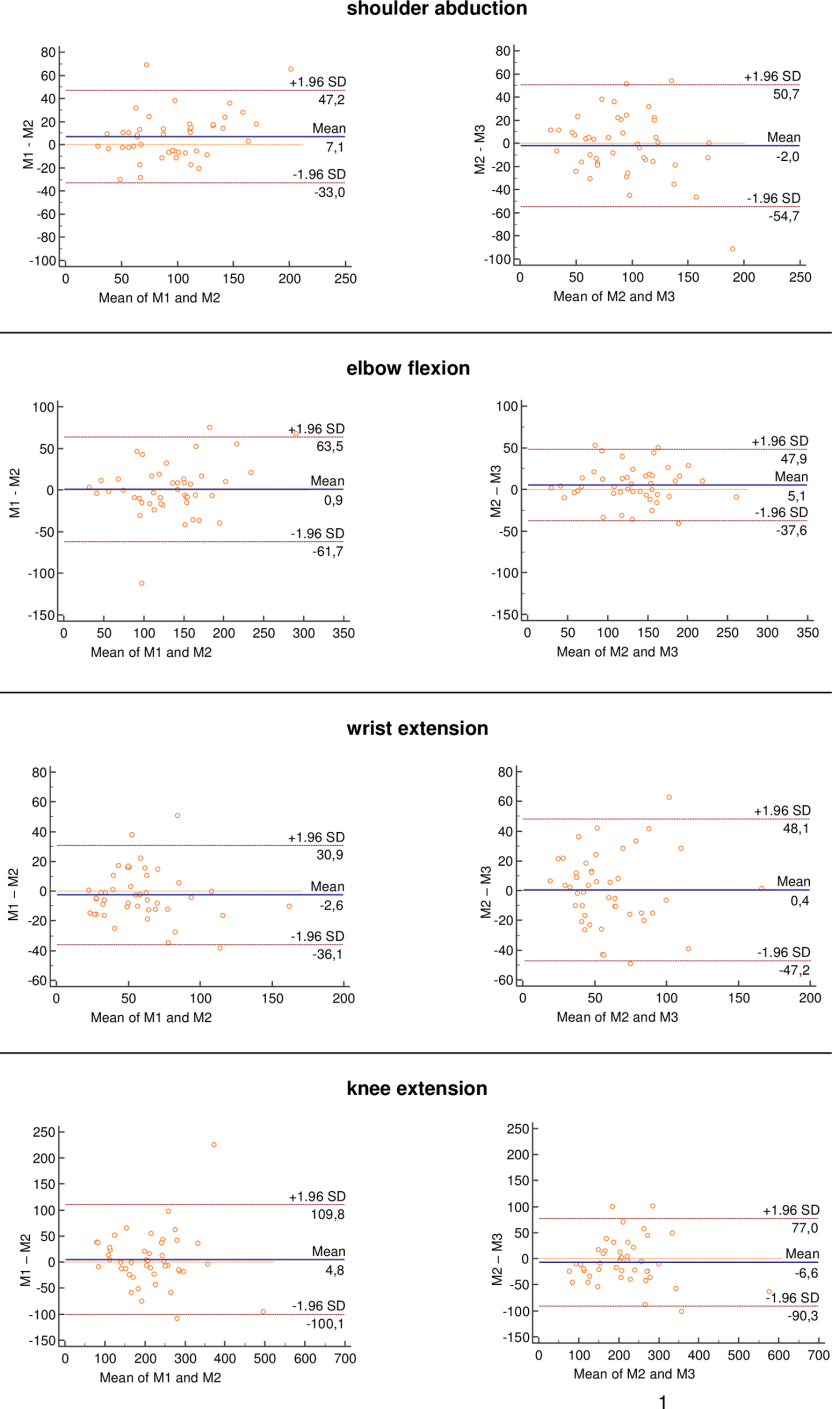
Bland and Altman plots of hand-held dynamometry (peak force). The blue continuous horizontal line shows the mean difference between tests. The dashed orange horizontal lines show the upper and lower 95% limits of agreement (bias ± 1.96 × standard deviation). Abbreviations: M: measurement, S_ABD: shoulder abduction, E_FLEX: elbow flexion, A_EXT: ankle extension, H_ABD: hip abduction, H_EXT: hip extension, K_EXT: knee extension, W_EXT: wrist extension, N_FLEX: neck flexion.

**Fig 4:**
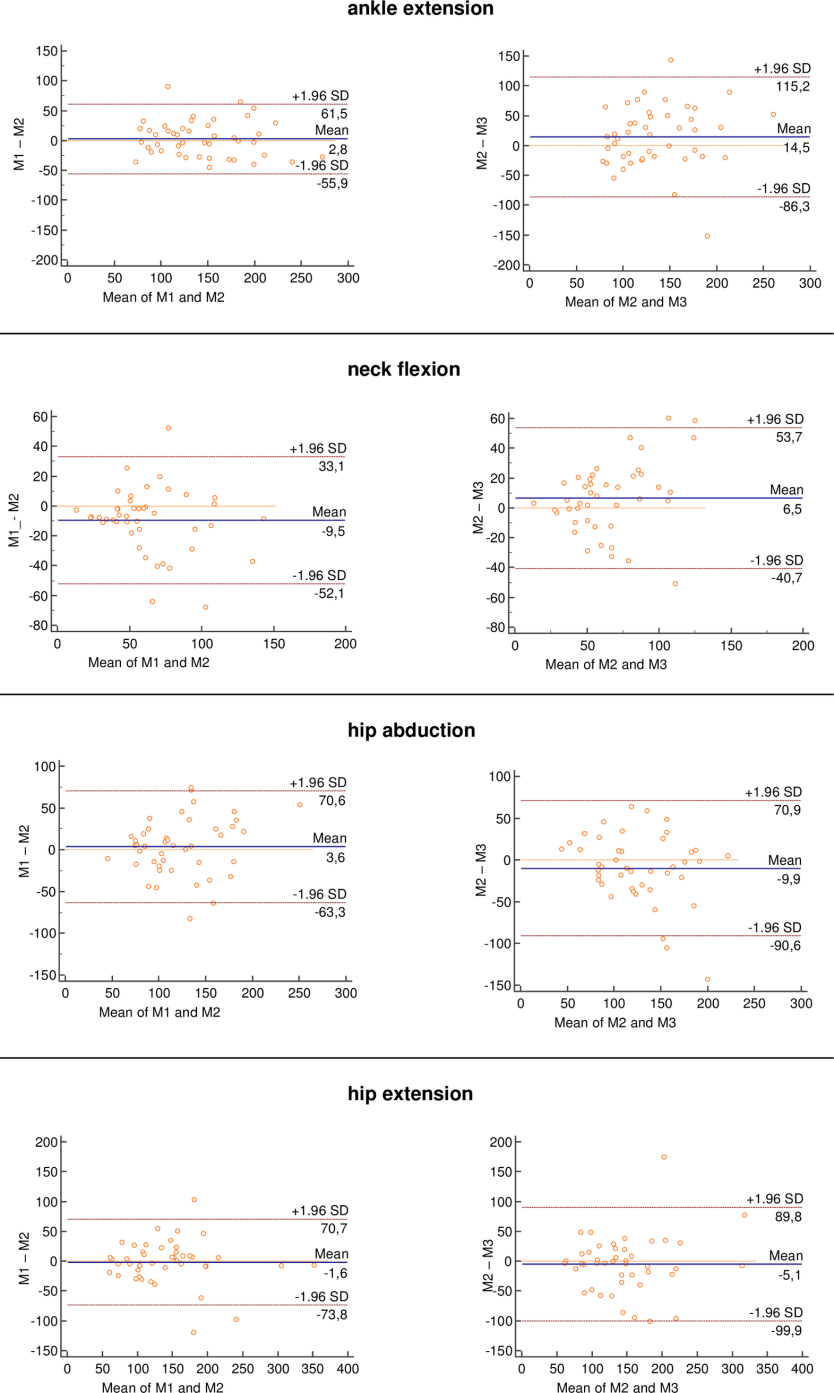
Bland and Altman plots of hand-held dynamometry (peak force). The blue continuous horizontal line shows the mean difference between tests. The dashed orange horizontal lines show the upper and lower 95% limits of agreement (bias ± 1.96 × standard deviation).Abbreviations: M: measurement, S_ABD: shoulder abduction, E_FLEX: elbow flexion, A_EXT: ankle extension, H_ABD: hip abduction, H_EXT: hip extension, K_EXT: knee extension, W_EXT: wrist extension, N_FLEX: neck flexion.

Bland Altman plots between M1 and M2 (intrarater) and between M2 and M3 (interrater) are shown for peak force (Fig 3 and Fig 4). For all comparisons, most of the data were within two standard deviations in the Bland-Altman plots. The plots illustrated small, but non-systematic errors between test and retest. Limits of agreement were always greater for intra-than for interrater reliability and visual inspection showed no tendency towards heteroscedasticity.

### Manual muscle testing

The results of the raw MMT8 score (M1, M2, M3, intrarater Kappa, and interrater Kappa) are presented in Table 5 (single muscle groups) and Table 6 (total score) and those from the graded score in Table 7.

**Table 5:** MMT8 single muscle groups at M1, M2, M3 plus intra- and interrater Kappa values (N = 46).

	M1			M2			M3			intratester	intertester
muscle groups	median	(min,max)	(25%, 75%)	median	(min, max)	(25%, 75%)	median	(min, max)	(25%, 75%)	Kappa	Kappa
S_ABD	9	(6,10)	(8, 10)	9	(6, 10)	(8, 10)	9	(5, 10)	(8, 10)	0.64^+++^	0.33^+^
E_FLEX	10	(6, 10)	(9, 10)	10*	(6, 10)	(9, 10)	10*	(8, 10)	(9, 10)	0.66^+++^	0.30^+^
W_EXT	10	(6, 10)	(9, 10)	10	(7, 10)	(9, 10)	10	(6, 10)	(9, 10)	0.53^++^	0.24^+^
K_EXT	10	(7,10)	(9, 10)	10	(6, 10)	(9, 10)	10	(7, 10)	(9, 10)	0.49^++^	0.08-
A_EXT	10	(7, 10)	(10, 10	10	(7, 10)	(9, 10)	10	(8, 10)	(10, 10)	0.35^+^	0.20^+^
N_FLEX	9	(3, 10)	(7,10)	8	(3, 10)	(7,10)	9**	(5, 10)	(7,10)	0.64^+++^	0.54^++^
H_ABD	9	(3, 10)	(7, 9)	9*	(3, 10)	(8, 9.5)	9*	(2, 10)	(7, 10)	0.66^+++^	0.44^++^
H_EXT	8	(1, 10)	(6, 9)	8*	(1, 10)	(7, 9)	8*	(2, 10)	(7, 10)	0.69^+++^	0.58^++^

M: measurement, min: minimum, max: maximum, S_ABD: shoulder abduction, E_FLEX: elbow flexion, A_EXT: ankle extension, H_ABD: hip abduction, H_EXT: hip extension, K_EXT: knee extension, W_EXT: wrist extension, N_FLEX: neck flexion

*1 missing values

** 2 missing values

^+^ fair reliability

^++^moderate reliability

^+++^substantial reliability

**Table 6:** MMT8 total score at M1, M2, M3 plus intra-and interrater reliability (N = 46).

	M1	M2	M3	intrarater reliability			interrater reliability		
	mean (±SD)	mean (±SD)	mean (±SD)	ICC (95%CI)	SEM SEM%	SDC SDC%	ICC (95%CI)	SEM SEM%	SDC SDC%
total score	70±8	71±7*	72±7*	0.94^++^ (0.90–0.97)	1.8 (2.5)	4.9 (6.9)	0.91^++^ (0.83–0.95)	2.2 (3.1)	6.2 (8.6)

M: measurement, SD: standard deviation, 95%CI: 95% confidence interval, ICC: Intraclass correlation coefficient

*3 missing values.

^++^excellent reliability

**Table 7:** MMT8 graded score at M1, M2, M3 plus intra- and interrater Kappa values (N = 46).

	M1				M2				M3				intratester	intertester
muscle groups	no_w %	mi_w %	mo_w %	s_w %	no_w %	mi_w %	mo_w %	s_w %	no_w %	mi_w %	mo_w %	s_w %	Kappa	Kappa
S_ABD	28.0	58.0	14.0	0.0	32.6	58.7	8.7	0.0	37.0	52.2	10.9	0.0	0.66^+++^	0.35^+^
E_FLEX	73.5	22.4	4.1	0.0	71.1*	22.2	6.7	0.0	73.9*	26.1	0.0	0.0	0.61^+++^	0.43^++^
W_EXT	63.3	32.7	4.1	0.0	69.6	30.4	0.0	0.0	71.7	26.1	2.2	0.0	0.55^++^	0.21^+^
K_EXT	62.0	38.0	0.0	0.0	71.7	26.1	2.2	0.0	60.9	39.1	0.0	0.0	0.49^++^	0.18-
A_EXT	82.0	18.0	0.0	0.0	73.9	26.1	0.0	0.0	80.4	19.6	0.0	0.0	0.37^+^	0.20^+^
N_FLEX	32.0	50.0	16.0	2.0	28.3	60.9	8.7	2.2	34.1**	45.5	20.5	0.0	0.68^+++^	0.58^++^
H_ABD	22.0	58.0	18.0	2.0	24.4*	64.4	8.9	2.2	46.7*	37.8	11.1	4.4	0.65^+++^	0.46^++^
H_EXT	12.0	62.0	22.0	4.0	22.2*	57.8	17.8	2.2	33.3*	48.9	15.6	2.2	0.63^+++^	0.65^+++^
Total score	4.1	89.8	6.1	0.0	4.5**	90.9	4.5	0.0	15.9**	79.5	4.5	0.0	0.88^++++^	0.42^++^

M: measurement, no_w: no weakness, mi_w: mild weakness, mo_w: moderate weakness, s_w: severe weakness, S_ABD: shoulder abduction, E_FLEX: elbow flexion, A_EXT: ankle extension, H_ABD: hip abduction, H_EXT: hip extension, K_EXT: knee extension, W_EXT: wrist extension, N_FLEX: neck flexion

*1 missing values

**2 missing values

^-^slight reliability

^+^fair reliability

^++^moderate reliability

^+++^substantial reliability

^++++^almost perfect reliability

**Fig 5:**
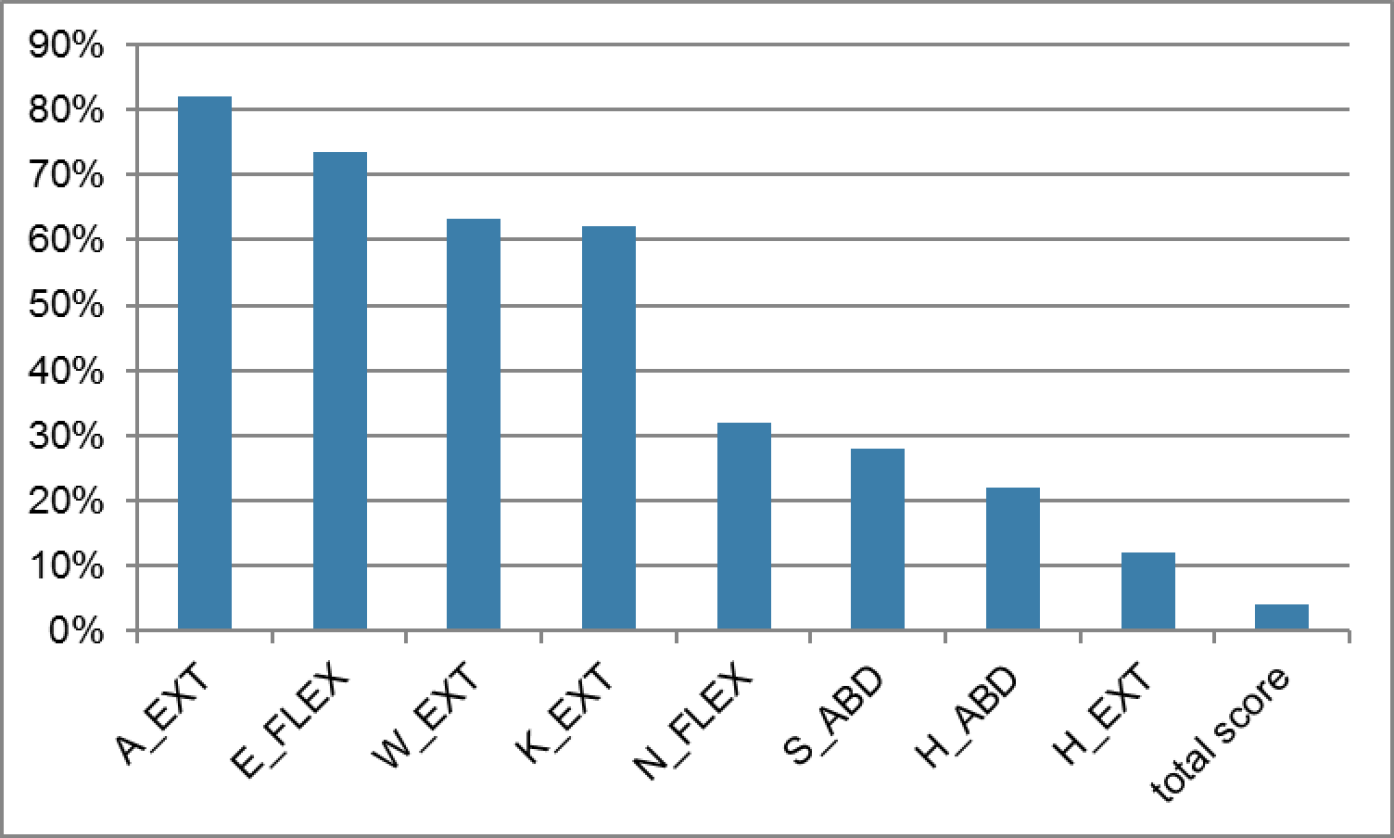
Ceiling effects of MMT8. All but one muscle group (hip extension) showed ceiling effects of 22 to 82%. Abbreviations: A_EXT: ankle extension, E_FLEX: elbow flexion, W_EXT: wrist extension, K_EXT: knee extension, N_FLEX: neck flexion, S_ABD: shoulder abduction, H_ABD: hip abduction, H_EXT: hip extension.

MMT-scores were between 1 and 10 for the weakest muscle group (hip extension) and between 7 and 10 for the strongest muscle group (knee extension and ankle extension). No differences between the measurements over time (M1, M2, M3) were seen (Wilcoxon, p≥0.003). All but one muscle group (hip extension) showed ceiling effects of 22 to 82% (Fig 5) with medians of the raw scores ranging from 8 to 10 points. The total raw score had no ceiling effect and varied from 46 to 80 with a mean of 70 points. The three muscle groups with the lowest score were neck flexion, hip abduction, and hip extension with moderate to severe weakness of 18%, 20%, 26%, respectively. Most of the participants had mild weakness (total graded score).

Intrarater reliability of the single muscle groups (raw as well as graded score) were substantial for shoulder abduction, elbow flexion, neck flexion, hip abduction, and hip extension (linear weighted Kappa varying from 0.61 to 0.69); moderate for wrist extension and knee extension (linear weighted Kappa varying from 0.49 to 0.53) and fair for ankle extension (linear weighted Kappa varying between 0.35 and 0.37). Interrater reliability (raw and graded scores) were moderate for neck flexion and hip abduction (linear weighted Kappa from 0.44 to 0.58); fair for shoulder abduction, wrist extension, and ankle extension (linear weighted Kappa varying from 0.20 to 0.35); and slight for knee extension (linear weighted Kappa of 0.08 and 0.18). Graded scores showed better Interrater reliability than row scores for elbow flexion (0.43 versus 0.3) and for hip extension (0.65 versus 0.59).

Intrarater and interrater reliability for total weakness score was substantial (0.88) and moderate (0.42), respectively. Intrarater and interrater reliability of the raw total score were excellent (ICC > 0.9 for both measures), and SEM and SDC% were 1.8 N and 6.9% and 2.2 N and 8.6%, respectively.

### Concordance between MMT and HHD

Analysis of inter-muscle-assessment-method showed low correlations for four muscle groups (wrist, knee, ankle and hip extension), moderate correlations for three muscle groups (hip abduction and elbow and neck flexion) and a good correlation for shoulder abduction between results obtained by the MMT8 and HHD, for both absolute force and force normalized to body weight (Table 8).

**Table 8:** Correlations (Spearman`s rho) between peak force (averaged and normalized) and MMT8 (N = 50).

muscle groups	averaged force	normalized force
S_ABD	0.85^+^	0.75^+^
E_FLEX	0.55^-^	0.54^-^
W_EXT	0.27	0.19
K_EXT	0.28	0.27
A_EXT	0.24	0.09
N_FLEX	0.62^-^	0.57^-^
H_ABD	0.61^-^	0.65^-^
H_EXT	0.28	0.38

S_ABD: shoulder abduction, E_FLEX: elbow flexion, A_EXT: ankle extension, H_ABD: hip abduction, H_EXT: hip extension, K_EXT: knee extension, W_EXT: wrist extension, N_FLEX: neck flexion, ^-^low correlation

^+^moderate correlation

**Fig 6:**
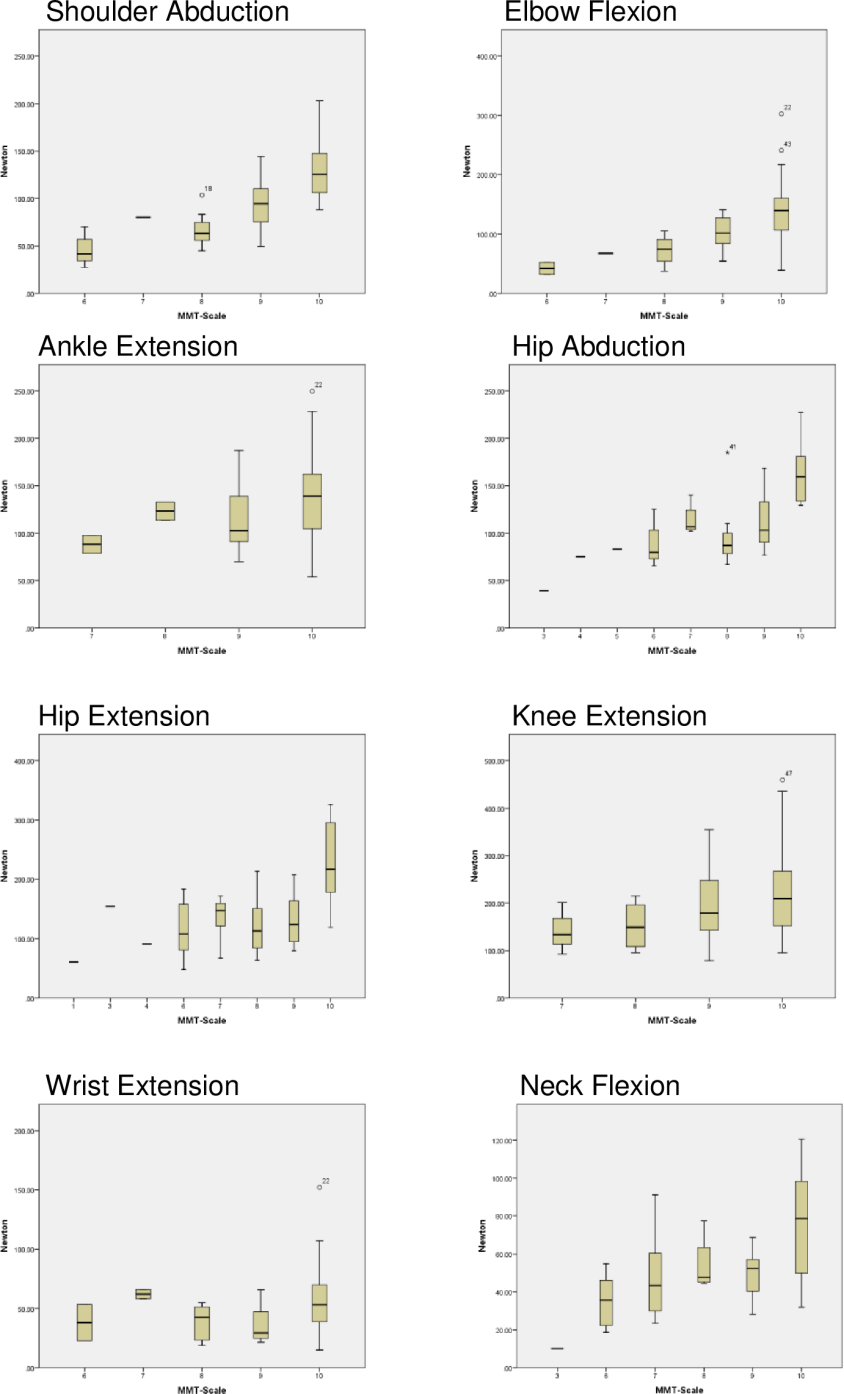
Muscle strength measured by hand-held dynamometry (in Newton) and MMT8 (MMT-Scale). The association between results from MMT8 and HHD are not consistent.

Fig 6 illustrates no consistent association between results from the MMT8 and the HHD in the different muscle groups. In elbow flexion, knee extension and neck flexion the median strength value is higher for a higher MMT score. However, the distribution of strength values for each muscle group showed a large range with considerable overlaps in the interquartile ranges. For the other four muscle groups (shoulder abduction, ankle extension, hip abduction, hip extension, wrist extension) the median strength value did not progressively increase between the consecutive score categories of MMT. Markedly, the median strength value is higher for grade seven than grade eight and nine in hip abduction, hip extension and wrist extension.

## Discussion

This study evaluated the intra- and interrater reliability of the MMT8 and a HHD, and the concordance between these two measures in a consecutively recruited convenience sample (n = 50) of people with myositis. In our sample, 76% of the participants were female. This gender distribution reflects the known higher prevalence of IM in females compared to males [31, 32].

The results of this study revealed excellent (ICC>0.7) intra- and-interrater reliability of the averaged peak force, except for the interrater reliability of ankle extension (ICC = 0.61). For peak force measurement, excellent ICCs were found for intrarater reliability for all muscle groups and the total score. Conversely, only three single muscle groups and the total score yielded excellent peak force interrater reliability scores. The SEMs and SDCs varied widely between single muscle groups. The SEMs% of the individual muscle groups ranged from 8 to 25% and the SDCs% from 23 to 78%. The SEMs% for the total score varied between 6 and 10% and the SDCs% between 16 and 27%. For the MMT8, the total score showed excellent intra- and interrater reliability (ICC>0.9), the single muscle group revealed Kappa values of 0.35–0.69 for intrarater reliability and values of 0.08–0.58 for interrater reliability, however, considerable ceiling effects (22–82%) were determined.

### Hand-held dynamometry

Our findings are in accordance with the findings from Stoll et al., who also reported excellent intra- and interrater reliability (ICCs intrarater: 0.88–0.98, ICCs interrater: 0.81–0.98) in seven people with myositis [17]. These results are only partially comparable, because different muscles groups were assessed. Neck flexion, shoulder elevation, elbow flexion and extension, hip flexion, and knee flexion and extension where evaluated by Stoll et al. while the muscle groups in our study were equal to those measured in the MMT8. Furthermore, no data about absolute reliability (measurement error) were reported by Stoll et al. [17]. Thus, it is not yet possible to compare the measurement errors of both studies and we cannot conclusively determine what measurement protocol leads to the optimal values to measure change in a patient’s strength values. Whether or not a measurement error is acceptable, depends on the amount of improvement or deterioration that one wants to detect [33]. The observed change in muscle strength must, therefore, be larger than the threshold of the SDC to ensure a real change in muscle strength. As ≥ 15% improvement in muscle strength is defined to be clinically relevant [18], an estimated SDC of ≤15% may be acceptable. The observed SDC measures in our study showed considerably higher values (SDCs between 29 and 65%) than the recommended 15%. However improvements of muscle strength varying between 38 and 62% are common [34, 35], therefore, dynamometry is capable to capture these improvements. These considerable improvements may be explained by the training principles of initial values, i.e. people with lowest level of fitness have greatest room for improvement [36].

As intrarater reliability is superior to interrater reliability, we recommend measurements to be performed by the same tester, a recommendation of particular importance when considering measurement error. Furthermore, reliability might be improved by using the average value of multiple measurements at each time point, instead of the peak force values [37]. We could confirm that ICCs and measurement errors were better for the averaged value of two performed measurements than for the maximum value. In clinical practice and research trial even three to four measurements were performed [9, 38].

**Fig 7:**
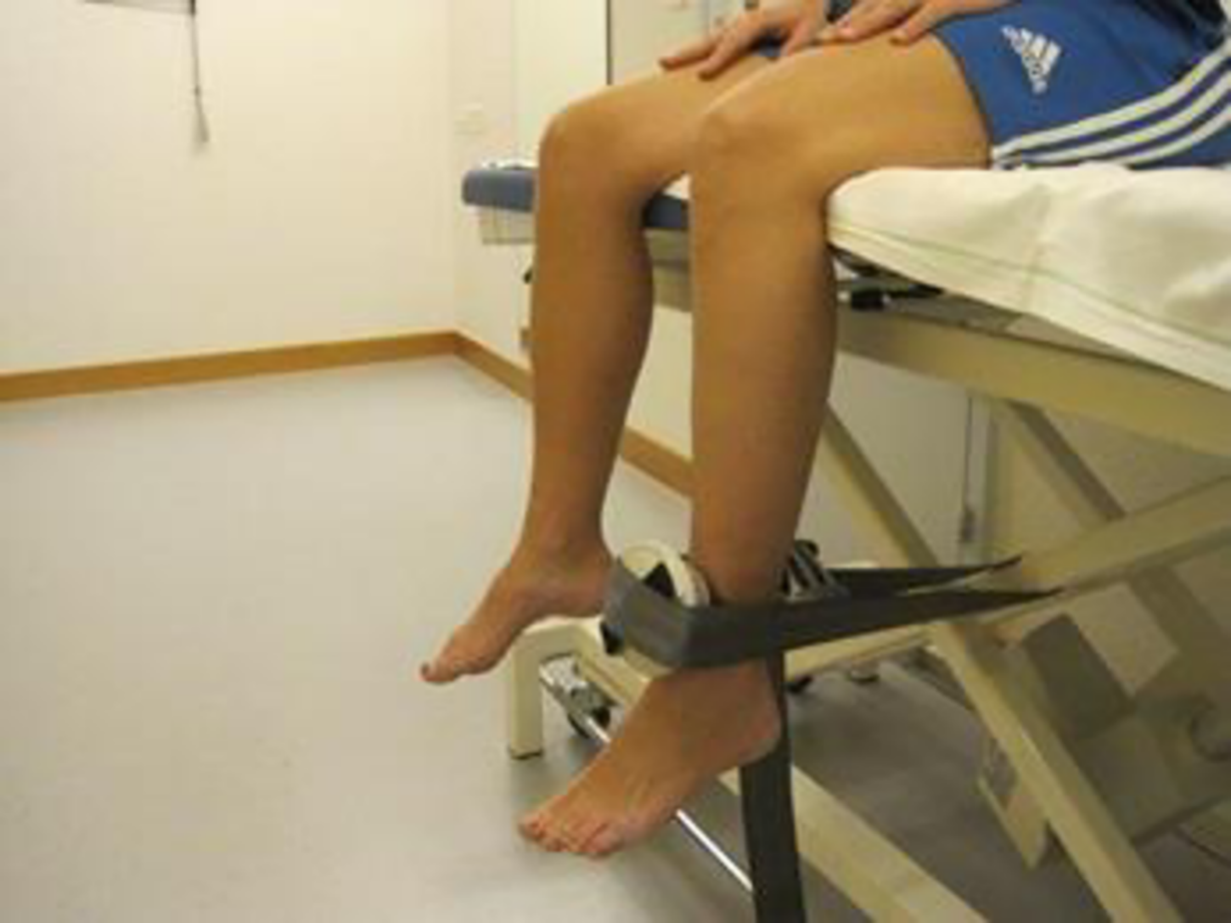
Measuring knee extension using a belt to stabilize the dynamometer. Standard body position of the patient and the device are shown with knees and hips flexed to 90°.

**Fig 8:**
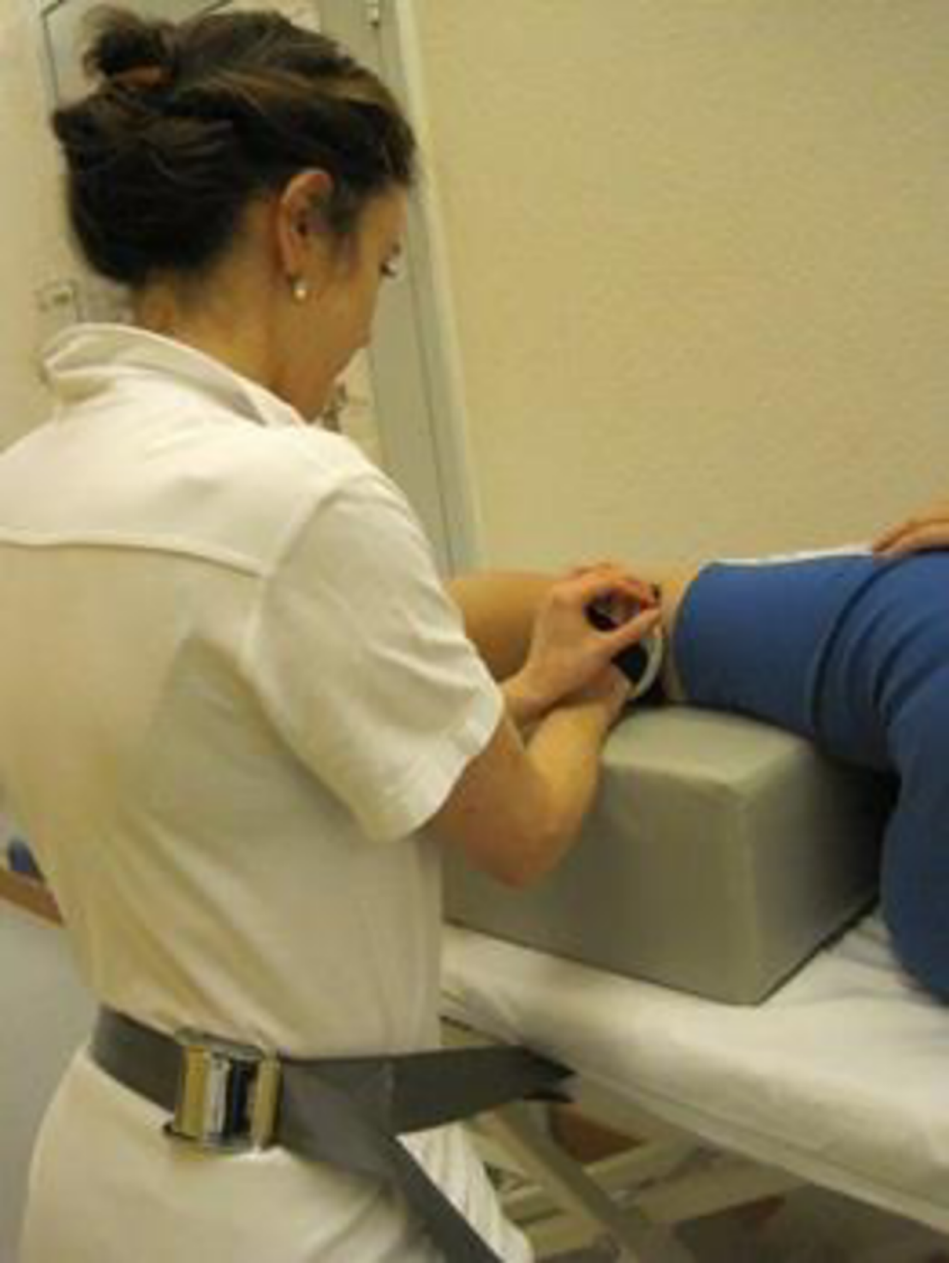
Measuring hip extension using a belt to stabilize the examiner. Standard body position of the patient and the device are shown with stretched hip and knees.

A well-known problem of hand held dynamometry is that the testers are often too weak to provide counterbalance to test certain lower extremity muscles [39]. Stone et al hypothesized that reliability was compromised by inadequate tester strength even in frail populations [40]. We tried to overcome this limitation by using a belt to stabilize the dynamometer or the examiner where this seemed necessary. When measuring knee extension, the dynamometer was always fixed with a belt (Fig 7). When measuring hip abduction and extension in strong participants the examiners stabilized themselves with a belt (Fig 8). Although measurement of knee extension could not be limited by the strength of the examiner, the reliability parameters were not superior for these measures compared to the other muscle groups. If the examiners’ strength were too low to assess actual strength, we would anticipate detection of a ceiling effect. As our data did not show any ceiling effect, we concluded that the force of the examiners was not a limiting factor.

### Manual muscle testing

Compared to other IM-trials, our participants showed relatively low muscle weakness. The median score of MMT8 in our sample was 10 to 50% higher than the score reported by Harris-Love et al. [19], and our total score exceeded the score reported by Rider et al. (87.5% versus 76.5%) [4]. We could confirm or even exceed known ceiling effects [5]. In seven out of eight muscle groups more than 20% of the included participants revealed the highest scores, which theoretically implies that these participants had no muscle weakness at the time of measurement. Conversely, Anderson et al. demonstrated a substantial number of participants (28–41%) classified with `normal`MMT values had muscle weakness following evaluation with isokinetic dynamometry. Therefore, the MMT cannot differentiate mild muscle weakness from normal muscle strength [41]. This finding was confirmed by Bohannon et al., who examined participants from four different studies with a manual muscle grade 5 (grade 5 of the Medical Research Council Scale equals 10 in the Kendal scale) and revealed that the highest grade encompassed a broad range of forces between 85N and 650 N. They concluded that MMT may lack the sensitivity to properly assess relatively strong muscle groups [42].

Whereas intrarater reliability of five single muscle groups was substantial, those of interrater reliability were only slight to moderate. One study, that evaluated reliability in adult people with myositis reported higher interrater reliability. The authors identified excellent interrater reliability for shoulder abduction, elbow flexion, knee extension and hip abduction, fair to good interrater reliability for hip extension, neck flexion and wrist extension, and poor for ankle extension [43]. This study included seven participants and used ICCs to calculate reliability, although MMT scores are ordinarily scaled. Therefore, these results should be interpreted with caution. The results of our study were partially in line with one report in which juvenile people with myositis (n = 10) were tested for intra-and interrater reliability. The intrarater reliability was also higher (Spearman`s rank correlation coefficient: 0.8) than interrater reliability (Kendall`s W: 0.72). In contrast to our study, the study of Rider et al. revealed acceptable interrater reliability [4]. Despite a detailed test protocol, standardised test environment, defined test order, and experienced and trained examiners, we could not reach satisfying interrater reliability for single muscle groups.

Nevertheless, intrarater as well as interrater reliability of the total score was excellent. These findings were supported by one report evaluating reliability in children with juvenile DM. The authors emphasized that it is important to use MMT summary scores, because the interrater reliability varies between individual muscle groups [44].

Absolute reliability could only be calculated for the total score. SDC and SDC per cent were lower for intrarater reliability (4.9 points, respectively 7%) than for interrater reliability (6.2 points, respectively 9%). A consortium of rheumatologists and neurologists has reached consensus that MMT8 should improve by ≥ 15% to classify adult people with PM/DM as improved [18]. According to our calculations the MMT8 total score is capable of capturing such improvements.

### Concordance between HHD and MMT8

Although the QMT and the MMT8 were both supposed to measure maximum isometric muscle strength the correlation for the majority of single muscle groups and the total score were only moderate or even worse. Additionally, graphical presentation of the data showed variable relationship between MMT and HHD. If MMT and HHD would measure the same construct of isometric muscle strength, we would expect that an increase in MMT scores correspond with an increase in the median of peak force of HHD and that the interquartile ranges between MMT scores would not overlap. In our data, only three muscle groups showed a constant increase in peak force and MMT-scores, but interquartile ranges were overlapping in all muscle groups. We could therefore confirm the variable relationship found by Noreau et al. for upper extremities [15]. In contrast to our results, previous studies reported good correlations (>0.7) between manual muscle test and HHD for knee extension [13, 14].

There are several possible explanations for these low correlations: First, high ceiling effects could be responsible for the low correlations. With the MMT8 no differences were seen for a considerable amount of participants (22–82%) whereas HHD gives different values for these participants. It seems to be difficult to detect and grade mild symmetrical muscle weakness with the MMT, partly because the examiner must consider the normal variation in strength in relation to age, weight, height, and gender [41]. Then, variations in the weight of the participant`s extremities, the force applied by the examiner, and the strength of the examiner could affect the subjective scoring of MMT8. Next, participant`s test position is different for MMT8 and HHD. While for the HHD, a gravity-neutralized position is needed, the MMT 8-test-position varies depending on the degree of weakness (from movement in horizontal plan to an antigravity position). For grades 5 and higher, participants have to hold the extremity against gravity and then the tester has to add pressure. The force needed to hold the extremity against gravity is not considered in scoring the MMT. Taken together, our results indicate that MMT does not measure the same parameter measured by HHD. Previous studies revealed that HHD is an appropriate method to assess isometric muscle strength compared with the gold standard isokinetic testing. Therefore, we conclude that MMT8 is an inadequate method to assess isometric muscle strength of individual muscle groups.

### Limitations and future research

This study had several limitations. First, a heterogeneous sample of people with myositis was included. Our participants suffer from different kind of myositis in different disease stages (acute, sub-acute and chronic). Due to inadequate sample size for a reliable subgroup analysis, we could not evaluate more homogeneous subsamples. Second, we did not record medications of our participants. Third, the measurements of this study were performed by two female examiners with several years of clinical experience and training in muscle strength assessment. Including more examiners in the reliability study would improve external validity of the results. Since strength assessment is exhausting for the people with myositis, we decided not to include more than two examiners in our study. Fourth, as no generally valid test protocol for HHD exits we developed our own measurement protocol, which hampers the comparison with other study results. Fifth, whilst MMT8-scores can be interpreted (severe, moderate, mild, no weakness), this is not possible with the HHD. However, individual strength values could be compared with normal reference values. Different authors published such reference values for shoulder abduction [20–22, 45–47], elbow flexion [20–22, 45–48], ankle extension [20–22, 45–48], hip abduction [20–22, 45, 47, 48], hip extension [47], knee extension [20–22, 45–48], wrist extension [20–22, 45–47], and neck flexion [22, 46, 47]. Because the published reference values were captured with different devices, in different test positions and with different placement of the devices, a direct comparison may not be adequate. None of these previous studies used the same device as we did and, to the best of our knowledge, there exists no reference values for this device. Bohannon et al emphasized that dynamometers should not be used interchangeable, because the magnitude of the force measured with two different devices differed significantly although they demonstrated good to high reliability and correlations [49]. Therefore, it is not possible to consider conclusively if a muscle group is weakened or not. Last, we did not include a gold standard for strength measurement.

To overcome these limitations future research should compile gender and age specific reference values for key muscles in people with myositis. Thereby the use of a generally accepted standardised protocol is important. These reference values may help to judge strength values of people with myositis. Furthermore, the validity of these muscle tests needs further investigation.

## Conclusion

The fact that the correlation between HHD and MMT8 is not satisfactory raises doubt as to whether the MMT8 measures the same construct (isometric strength) as HHD. The MMT8 total score is a reliable and time efficient assessment to consider general muscle weakness in people with myositis. However, since only the total score of MMT8 showed good reliability parameters MMT8 should not be used to evaluate changes (either improvement or deterioration) in single muscle groups of people with myositis. On the contrary, HHD could be recommended to evaluate isometric muscle strength of single muscle groups in people with myositis if the following important aspects are considered: examiners are experienced and trained in muscle testing, a standardised protocol is followed, a belt to stabilize examiner or the device is used, and the average of at least two measures is applied.

## Supporting information

S1 Appendix Supporting information-data_file_MMT8_HHD. (XLSX)

## Abbreviations

IM: Inflammatory myopathy
DM: dermatomyositis
PM: polmyositis
MMT: manual muscle testing
MMT 8: short version of the of the original MMT
HHD: hand-held dynamometry
SEM: standard error of measurement
SDC: smallest detectable change
ICC: intraclass correlation coefficient
N: Newton
M1: measurement 1
M2: measurement 2
M3: measurement 3

## References

[1] Greenberg SA. Inflammatory myopathies: evaluation and management. Semin Neurol. 2008;28(2):241–9. Epub 2008/03/21. doi: 10.1055/s-2008-1062267 . 18351525 10.1055/s-2008-1062267

[2] Dalakas MC. Polymyositis, dermatomyositis and inclusion-body myositis. N Engl J Med. 1991;325(21):1487–98. Epub 1991/11/21. doi: 10.1056/NEJM199111213252107 . 1658649 10.1056/NEJM199111213252107

[3] Bohan A, Peter JB. Polymyositis and dermatomyositis (second of two parts). N Engl J Med. 1975;292(8):403–7. Epub 1975/02/20. doi: 10.1056/NEJM197502202920807 . 1089199 10.1056/NEJM197502202920807

[4] Rider LG, Koziol D, Giannini EH, Jain MS, Smith MR, Whitney-Mahoney K, et al Validation of manual muscle testing and a subset of eight muscles for adult and juvenile idiopathic inflammatory myopathies. Arthritis Care Res (Hoboken). 2010;62(4):465–72. Epub 2010/04/15. doi: 10.1002/acr.20035 ; PubMed Central PMCID: PMC2924143. 20391500 10.1002/acr.20035 PMC2924143

[5] Rider LG, Werth VP, Huber AM, Alexanderson H, Rao AP, Ruperto N, et al Measures of adult and juvenile dermatomyositis, polymyositis, and inclusion body myositis: Physician and Patient/Parent Global Activity, Manual Muscle Testing (MMT), Health Assessment Questionnaire (HAQ)/Childhood Health Assessment Questionnaire (C-HAQ), Childhood Myositis Assessment Scale (CMAS), Myositis Disease Activity Assessment Tool (MDAAT), Disease Activity Score (DAS), Short Form 36 (SF-36), Child Health Questionnaire (CHQ), physician global damage, Myositis Damage Index (MDI), Quantitative Muscle Testing (QMT), Myositis Functional Index-2 (FI-2), Myositis Activities Profile (MAP), Inclusion Body Myositis Functional Rating Scale (IBMFRS), Cutaneous Dermatomyositis Disease Area and Severity Index (CDASI), Cutaneous Assessment Tool (CAT), Dermatomyositis Skin Severity Index (DSSI), Skindex, and Dermatology Life Quality Index (DLQI). Arthritis Care Res (Hoboken). 2011;63 Suppl 11:S118–57. Epub 2012/05/25. doi: 10.1002/acr.20532 . 22588740 10.1002/acr.20532 PMC3748930

[6] Bohan A, Peter JB. Polymyositis and dermatomyositis (first of two parts). N Engl J Med. 1975;292(7):344–7. Epub 1975/02/13. doi: 10.1056/NEJM197502132920706 . 1090839 10.1056/NEJM197502132920706

[7] Rider LG. Outcome assessment in the adult and juvenile idiopathic inflammatory myopathies. Rheum Dis Clin North Am. 2002;28(4):935–77. Epub 2003/01/01. PubMed . 12506779 10.1016/s0889-857x(02)00027-3

[8] Sultan SM. The assessment and importance of disease activity versus disease damage in patients with inflammatory myopathy. Curr Rheumatol Rep. 2003;5(6):445–50. Epub 2003/11/12. PubMed . 14609489 10.1007/s11926-003-0055-z

[9] Knols RH, Aufdemkampe G, de Bruin ED, Uebelhart D, Aaronson NK. Hand-held dynamometry in patients with haematological malignancies: measurement error in the clinical assessment of knee extension strength. BMC musculoskeletal disorders. 2009;10:31 Epub 2009/03/11. doi: 10.1186/1471-2474-10-31 ; PubMed Central PMCID: PMCPMC2662793. 19272149 10.1186/1471-2474-10-31 PMC2662793

[10] Mentiplay BF, Perraton LG, Bower KJ, Adair B, Pua YH, Williams GP, et al Assessment of Lower Limb Muscle Strength and Power Using Hand-Held and Fixed Dynamometry: A Reliability and Validity Study. PloS one. 2015;10(10):e0140822 Epub 2015/10/29. doi: 10.1371/journal.pone.0140822 ; PubMed Central PMCID: PMCPmc4624940. 26509265 10.1371/journal.pone.0140822 PMC4624940

[11] Stark T, Walker B, Phillips JK, Fejer R, Beck R. Hand-held dynamometry correlation with the gold standard isokinetic dynamometry: a systematic review. PM & R: the journal of injury, function, and rehabilitation. 2011;3(5):472–9. Epub 2011/05/17. doi: 10.1016/j.pmrj.2010.10.025 . 21570036 10.1016/j.pmrj.2010.10.025

[12] Kolber MJ, Cleland JA. Strength testing using hand-held dynamometry. Physical Therapy Reviews. 2005;10(2):99–112. doi: 10.1179/108331905X55730

[13] Bohannon RW. Measuring knee extensor muscle strength. American journal of physical medicine & rehabilitation / Association of Academic Physiatrists. 2001;80(1):13–8. Epub 2001/01/04. PubMed . 11138949 10.1097/00002060-200101000-00004

[14] Hayes KW, Falconer J. Reliability of hand-held dynamometry and its relationship with manual muscle testing in patients with osteoarthritis in the knee. The Journal of orthopaedic and sports physical therapy. 1992;16(3):145–9. Epub 1992/01/01. doi: 10.2519/jospt.1992.16.3.145 . 18796764 10.2519/jospt.1992.16.3.145

[15] Noreau L, Vachon J. Comparison of three methods to assess muscular strength in individuals with spinal cord injury. Spinal cord. 1998;36(10):716–23. Epub 1998/11/04. PubMed . 9800275 10.1038/sj.sc.3100646

[16] Aitkens S, Lord J, Bernauer E, Fowler WM Jr., Lieberman JS, Berck P. Relationship of manual muscle testing to objective strength measurements. Muscle & nerve. 1989;12(3):173–7. Epub 1989/03/01. doi: 10.1002/mus.880120302 . 2725546 10.1002/mus.880120302

[17] Stoll T, Bruhlmann P, Stucki G, Seifert B, Michel BA. Muscle strength assessment in polymyositis and dermatomyositis evaluation of the reliability and clinical use of a new, quantitative, easily applicable method. J Rheumatol. 1995;22(3):473–7. Epub 1995/03/01. PubMed . 7783064

[18] Rider LG, Giannini EH, Harris-Love M, Joe G, Isenberg D, Pilkington C, et al Defining Clinical Improvement in Adult and Juvenile Myositis. J Rheumatol. 2003;30(3):603–17. Epub 2003/03/01. PubMed . 12610824

[19] Harris-Love MO, Shrader JA, Koziol D, Pahlajani N, Jain M, Smith M, et al Distribution and severity of weakness among patients with polymyositis, dermatomyositis and juvenile dermatomyositis. Rheumatology (Oxford). 2009;48(2):134–9. Epub 2008/12/17. doi: 10.1093/rheumatology/ken441 ; PubMed Central PMCID: PMC2634286. 19074186 10.1093/rheumatology/ken441 PMC2634286

[20] Andrews AW, Thomas MW, Bohannon RW. Normative values for isometric muscle force measurements obtained with hand-held dynamometers. Phys Ther. 1996;76(3):248–59. Epub 1996/03/01. PubMed . 8602410 10.1093/ptj/76.3.248

[21] Bohannon RW. Reference values for extremity muscle strength obtained by hand-held dynamometry from adults aged 20 to 79 years. Arch Phys Med Rehabil. 1997;78(1):26–32. Epub 1997/01/01. PubMed . 9014953 10.1016/s0003-9993(97)90005-8

[22] van der Ploeg RJ, Fidler V, Oosterhuis HJ. Hand-held myometry: reference values. Journal of Neurology, Neurosurgery & Psychiatry. 1991;54(3):244–7. doi: 10.1136/jnnp.54.3.244 10.1136/jnnp.54.3.244 PMC1014394 2030353

[23] Vet HCWd. Measurement in Medicine: A Practical Guide: Cambridge: Cambridge University Press; 2011. Online-Ressource p.

[24] Streiner DL, Norman GR. Health measurement scales: a practical guide to their development and use 4th ed. ed: Oxford: Oxford University Press; 2008. 431 S. p.

[25] Fleiss JL. Reliability of Measurement The Design and Analysis of Clinical Experiments: John Wiley & Sons, Inc.; 1999 p. 1–32.

[26] de Vet HC, Terwee CB, Knol DL, Bouter LM. When to use agreement versus reliability measures. J Clin Epidemiol. 2006;59(10):1033–9. Epub 2006/09/19. doi: 10.1016/j.jclinepi.2005.10.015 . 16980142 10.1016/j.jclinepi.2005.10.015

[27] Bland JM, Altman DG. Statistical methods for assessing agreement between two methods of clinical measurement. Lancet. 1986;1(8476):307–10. Epub 1986/02/08. PubMed . 2868172

[28] Terwee CB, Bot SD, de Boer MR, van der Windt DA, Knol DL, Dekker J, et al Quality criteria were proposed for measurement properties of health status questionnaires. J Clin Epidemiol. 2007;60(1):34–42. Epub 2006/12/13. doi: 10.1016/j.jclinepi.2006.03.012 . 17161752 10.1016/j.jclinepi.2006.03.012

[29] Landis JR, Koch GG. The Measurement of Observer Agreement for Categorical Data. Biometrics. 1977;33(1):159–74. doi: 10.2307/2529310 843571

[30] Siegel S, Castellan J. Nonparametric Statistics for the Behavioral Sciences. 2nd (Januar 1988) ed. Boston Massachusetts: McGraw-Hill Inc.; 1988.

[31] Mastaglia FL, Phillips BA. Idiopathic inflammatory myopathies: epidemiology, classification, and diagnostic criteria. Rheum Dis Clin North Am. 2002;28(4):723–41. Epub 2003/01/04. PubMed . 12510664 10.1016/s0889-857x(02)00021-2

[32] Meyer A, Meyer N, Schaeffer M, Gottenberg JE, Geny B, Sibilia J. Incidence and prevalence of inflammatory myopathies: a systematic review. Rheumatology (Oxford). 2015;54(1):50–63. Epub 2014/07/30. doi: 10.1093/rheumatology/keu289 . 25065005 10.1093/rheumatology/keu289

[33] Scholtes VA, Terwee CB, Poolman RW. What makes a measurement instrument valid and reliable? Injury. 2011;42(3):236–40. Epub 2010/12/15. doi: 10.1016/j.injury.2010.11.042 . 21145544 10.1016/j.injury.2010.11.042

[34] Varju C, Petho E, Kutas R, Czirjak L. The effect of physical exercise following acute disease exacerbation in patients with dermato/polymyositis. Clin Rehabil. 2003;17(1):83–7. Epub 2003/03/06. doi: 10.1191/0269215503cr572oa . 12617382 10.1191/0269215503cr572oa

[35] Hengstman GJ, van den Hoogen FH, Barrera P, Netea MG, Pieterse A, van de Putte LB, et al Successful treatment of dermatomyositis and polymyositis with anti-tumor-necrosis-factor-alpha: preliminary observations. European neurology. 2003;50(1):10–5. Epub 2003/06/26. doi: 70852. doi: 10.1159/000070852 . 12824706 10.1159/000070852

[36] Baschung Pfister P, de Bruin E, Tobler-Ammann B, Maurer B, Knols R. The relevance of applying exercise training principles when designing therapeutic interventions for patients with inflammatory myopathies: a systematic review. Rheumatol Int. 2015;35(10):1641–54. doi: 10.1007/s00296-015-3343-9 26271469 10.1007/s00296-015-3343-9

[37] de Winter AF, Heemskerk MA, Terwee CB, Jans MP, Deville W, van Schaardenburg DJ, et al Inter-observer reproducibility of measurements of range of motion in patients with shoulder pain using a digital inclinometer. BMC musculoskeletal disorders. 2004;5:18 Epub 2004/06/16. doi: 10.1186/1471-2474-5-18 ; PubMed Central PMCID: PMCPMC434511. 15196309 10.1186/1471-2474-5-18 PMC434511

[38] Sole G, Hamren J, Milosavljevic S, Nicholson H, Sullivan SJ. Test-retest reliability of isokinetic knee extension and flexion. Arch Phys Med Rehabil. 2007;88(5):626–31. Epub 2007/05/01. doi: 10.1016/j.apmr.2007.02.006 . 17466732 10.1016/j.apmr.2007.02.006

[39] Rider LG, Giannini EH, Brunner HI, Ruperto N, James-Newton L, Reed AM, et al International consensus on preliminary definitions of improvement in adult and juvenile myositis. Arthritis Rheum. 2004;50(7):2281–90. Epub 2004/07/13. doi: 10.1002/art.20349 . 15248228 10.1002/art.20349

[40] Stone CA, Nolan B, Lawlor PG, Kenny RA. Hand-held dynamometry: tester strength is paramount, even in frail populations. Journal of rehabilitation medicine. 2011;43(9):808–11. Epub 2011/08/10. doi: 10.2340/16501977-0860 . 21826388 10.2340/16501977-0860

[41] Andersen H, Jakobsen J. A comparative study of isokinetic dynamometry and manual muscle testing of ankle dorsal and plantar flexors and knee extensors and flexors. European neurology. 1997;37(4):239–42. Epub 1997/01/01. doi: 10.1159/000117450 . 9208265 10.1159/000117450

[42] Bohannon RW, Corrigan D. A broad range of forces is encompassed by the maximum manual muscle test grade of five. Perceptual and motor skills. 2000;90(3 Pt 1):747–50. Epub 2000/07/07. doi: 10.2466/pms.2000.90.3.747 . 10883753 10.2466/pms.2000.90.3.747

[43] Isenberg DA, Allen E, Farewell V, Ehrenstein MR, Hanna MG, Lundberg IE, et al International consensus outcome measures for patients with idiopathic inflammatory myopathies. Development and initial validation of myositis activity and damage indices in patients with adult onset disease. Rheumatology (Oxford). 2004;43(1):49–54. Epub 2003/07/18. doi: 10.1093/rheumatology/keg427 . 12867580 10.1093/rheumatology/keg427

[44] Jain M, Smith M, Cintas H, Koziol D, Wesley R, Harris-Love M, et al Intra-rater and inter-rater reliability of the 10-point Manual Muscle Test (MMT) of strength in children with juvenile idiopathic inflammatory myopathies (JIIM). Physical & occupational therapy in pediatrics. 2006;26(3):5–17. Epub 2006/09/13. PubMed . 16966313

[45] Backman E, Johansson V, Hager B, Sjoblom P, Henriksson KG. Isometric muscle strength and muscular endurance in normal persons aged between 17 and 70 years. Scandinavian journal of rehabilitation medicine. 1995;27(2):109–17. Epub 1995/06/01. PubMed . 7569820

[46] Meldrum D, Cahalane E, Conroy R, Fitzgerald D, Hardiman O. Maximum voluntary isometric contraction: reference values and clinical application. Amyotrophic lateral sclerosis: official publication of the World Federation of Neurology Research Group on Motor Neuron Diseases. 2007;8(1):47–55. Epub 2007/03/17. doi: 10.1080/17482960601012491 . 17364436 10.1080/17482960601012491

[47] Stoll T, Huber E, Seifert B, Michel BA, Stucki G. Maximal isometric muscle strength: normative values and gender-specific relation to age. Clin Rheumatol. 2000;19(2):105–13. Epub 2000/05/03. PubMed . 10791620 10.1007/s100670050026

[48] McKay MJ, Baldwin JN, Ferreira P, Simic M, Vanicek N, Burns J. Normative reference values for strength and flexibility of 1,000 children and adults. Neurology. 2017;88(1):36–43. Epub 2016/11/25. doi: 10.1212/WNL.0000000000003466 ; PubMed Central PMCID: PMCPmc5200854. 27881628 10.1212/WNL.0000000000003466 PMC5200854

[49] Bohannon RW. Comparability of force measurements obtained with different strain gauge hand-held dynamometers. The Journal of orthopaedic and sports physical therapy. 1993;18(4):564–7. Epub 1993/10/01. doi: 10.2519/jospt.1993.18.4.564 . 8220416 10.2519/jospt.1993.18.4.564

